# Sleep Disturbances and Co-sleeping in Italian Children and Adolescents with Autism Spectrum Disorder

**DOI:** 10.1007/s10803-024-06507-y

**Published:** 2024-08-08

**Authors:** Serena Scarpelli, Deny Menghini, Valentina Alfonsi, Francesca Giumello, Ludovica Annarumma, Maurizio Gorgoni, Giovanni Valeri, Mariella Pazzaglia, Luigi De Gennaro, Stefano Vicari

**Affiliations:** 1 Department of Psychology, Sapienza University of Rome, Rome, Italy; 2 Child and Adolescent Neuropsychiatry Unit, Bambino Gesù Children’s Hospital, IRCCS, Rome, Italy; 3 IRCCS Fondazione Santa Lucia, Rome, Italy; 4 Department of Life Science and Public Health, Catholic University, 00168 Rome, Italy

**Keywords:** Sleep, Autism, Insomnia, Neurodevelopmental disorder, Co-sleeping

## Abstract

The current study aimed (1) to provide an analysis of the frequency and prevalence of sleep disturbances in a large Italian sample of children and adolescents with ASD, detecting specific predictors of the presence/absence of sleep disorders, (2) to examine the phenomenon of co-sleeping within a subgroup of participants with ASD. A total of 242 children and adolescents with ASD (194 males, mean age 5.03 ± 3.15 years) were included. After the diagnostic procedure, caregivers were requested to complete the Sleep Disturbance Scale for Children (SDSC) to assess sleep disorders among participants. The presence of co-sleeping was investigated in a subgroup of 146 children and adolescents with ASD. An elevated or clinically relevant global score for sleep disorders (≥ 60) was found in 33% of participants. The most prevalent sleep disorder in our group was related to difficulties with sleep onset and sleep maintenance (~ 41% of cases). Sleep disturbances were predicted by higher intelligence quotient (IQ)/developmental quotient (DQ), increased internalizing problems, and elevated parental stress. The subgroup of participants engaged in co-sleeping (N = 87) were younger and had lower IQ/DQ scores, reduced adaptive functioning, and diminished psychological wellbeing than the non-co-sleeping group. Our findings are consistent with the current literature highlighting that insomnia is the most widespread sleep problem associated with ASD. The relationship between IQ/DQ and sleep alterations is a crucial topic that deserves additional research. Future studies should assess sleep by objective measures such as EEG topography to better understand the mechanisms underlying sleep alterations in this neurodevelopmental disorder.

## Introduction

Neurodevelopmental disorders are often associated with sleep alterations (Gorgoni et al., 2020) and sleep disturbances (Kamara & Beacuchaine, 2020). A recent systematic review (Carmassi et al., 2019) highlighted that in addition to specific core features (i.e., repetitive behaviors, restricted interests, difficulties in social interactions, altered sensory processing, and insistence on sameness), sleep problems are often experienced by individuals with autism spectrum disorder (ASD). Despite this, prevalence data show high variability across studies (Carmassi et al., 2019). Some findings report a prevalence between 64 and 93% (Irwanto et al., 2016; May et al., 2015; Rzepecka et al., 2011; Taira et al., 1998; Tudor et al., 2015; Wiggs & Stores, 2004). Specifically, short sleep duration, poor sleep quality, and delayed circadian rhythms are frequent in children and adolescents with ASD (for review, see Carmassi et al., 2019).

Recent evidence confirms that parental reports of insomnia symptoms and bedtime resistance are particularly common in children with ASD (Bernardi et al., 2023; Galli et al., 2022). Immediate-release or extended-release melatonin was often prescribed for these sleep problems, with treatment lasting more than a year. Sleep hygiene protocols (i.e., bedtime routines, appropriate sleep timing, relaxation techniques, and healthy diet) are also recommended, although Bernardi and coworkers (2023) highlighted that only a percentage ranging from 20 to 30% of parents reported the effectiveness of these practices.

Other sleep disturbances in autism have been identified, such as parasomnias (Bernardi et al., 2023; Tudor et al., 2012) and excessive daytime sleepiness (Fadini et al., 2015), both of which showed an association with behavioral problems and ASD severity (Fadini et al., 2015; Tudor et al., 2012).

Actually, several findings revealed that sleep difficulties can exacerbate ASD symptoms (Adams et al., 2014; Schreck et al., 2004; Schreck & Richdale, 2020; Tudor et al., 2012; Veatch et al., 2017; Whelan et al., 2022). Children with ASD who experience sleep problems tend to report greater deficits in social skills than children with ASD who do not experience sleep problems (Schreck & Richdale, 2020). Consistent with this, Veatch et al. (2017) found that sleep duration appeared to be negatively correlated with severity scores for social/communication impairments and restricted and repetitive behaviors. Accordingly, a recent review highlighted the association between sleep quality and social functioning, suggesting a potential bidirectional relationship (Whelan et al., 2022). Furthermore, an interaction between altered sensory processing and sleep disorders has been suggested (Deliens & Peigneux, 2019). Recent cross-sectional findings showed that movement sensitivity and auditory filtering were positively and negatively correlated with the total score for sleep disorders, respectively (Deliens & Peigneux, 2019). However, other studies have not found a significant association between sleep and the severity of core ASD symptoms (Anders et al., 2012; Mutluer et al., 2016). In fact, Mutluer and collaborators (2016) compared sleep-related problems (i.e., snoring, breathing symptoms, periodic sleep movement disorder, insomnia, sleepiness, and other sleep problems) between different ASD severity subgroups (i.e., mild-moderate and severe subtypes) and found no differences. Also, actigraphic sleep measures (i.e., sleep efficiency, sleep onset latency, number of awakenings, and wakefulness after sleep onset duration) were not significantly associated with daytime behavior problems in ASD (Anders et al., 2012).

The mechanisms underlying sleep problems in ASD remain unclear. Sleep disturbances may result from intrinsic biological/genetic abnormalities that alter the architecture or biochemistry of the sleeping brain, psychological/behavioral characteristics related to ASD symptoms, or environmental factors including poor sleep hygiene practices (Richdale & Schreck, 2009). Interestingly, converging evidence has shown dysregulation of cortisol (Corbett et al., 2009; Tomarken et al., 2015) and melatonin levels (Kulman et al., 2000; Nir et al., 1995), which may contribute to circadian rhythm alterations and insomnia symptoms in ASD, particularly in adolescents who are more likely to report delayed sleep phase syndrome (Oyane and Bjorvatn, 2005).

Although heterogeneous, electrophysiological findings in patients with ASD revealed altered slow wave activity (SWA) and spindles, which may indicate impaired thalamocortical pathways and anatomical/functional connectivity during sleep (Gorgoni et al., 2020).

Although not always consistent, some research has suggested that intra-individual factors may be associated with sleep problems in ASD. Some studies have found a significant relationship between cognitive functioning and sleep disturbances (e.g., Bruni et al., 2007; Limoges et al., 2013). For example, Bruni et al. (2007) provided a Cycling Alternating Pattern analysis showing that children with ASD had a decreased A1 index, (i.e., fewer phases of stable, restorative deep sleep Slow Wave Sleep, SWS), and increased A2 and A3 indexes (i.e., more frequent transitions and unstable phases during light sleep), compared to controls. In other words, they found greater cortical arousal in the ASD group. In contrast, subjects with Asperger syndrome showed patterns similar to those found in typically developing children (Bruni et al., 2007). This group showed a positive correlation between verbal intelligence quotient (IQ) and the A1 index during deep sleep, while the percentage of A2—an index of higher sleep fragmentation—negatively correlated with full-scale IQ, verbal IQ, and performance IQ (Bruni et al., 2007). Moreover, in adults with ASD, some findings revealed a negative correlation between sleep spindles and the number of trials needed to learn a procedural memory task (Limoges et al., 2013).

Additionally, psychological conditions such as anxiety symptoms have been linked to sleep disturbances in autism (Mazurek & Petroski, 2015). A study encompassing a large sample of individuals aged 2 to 18 with ASD revealed that anxiety was associated with various sleep issues including bedtime resistance, delayed sleep onset, short sleep duration, and intra-sleep awakenings (Mazurek & Petroski, 2015).

It should be noted that medical comorbidities are very common in individuals with ASD and these conditions may also affect sleep patterns (Al-Beltagi, 2021). Approximately 30% of individuals with ASD have EEG abnormalities or epileptic discharges (Accardo & Malow, 2015). In addition, gastrointestinal problems and altered immune function may be associated with sleep disturbances (Al-Beltagi, 2021).

Sleep disruption in ASD has been associated with increased maternal distress and parental sleep difficulties, as well as poor caregiver quality of life (Doo & Wing, 2006; Devnani & Hedge, 2015). Notably, “co-sleeping” has been described as a common habit among children and adolescents with ASD (Köse et al., 2017). Co-sleeping is defined as an “intentional” or “reactive” practice in which children and parents sleep together during the night. This practice includes 'bed-sharing' (sharing the same bed for sleeping) and 'room-sharing' (sharing the same room) (Mileva-Seitz et al., 2017). The overall prevalence of co-sleeping is difficult to determine. Rates of co-sleeping vary considerably between cultures and over different time periods (Köse et al., 2017). Köse et al. (2017) found that co-sleeping with a parent and sleep disturbances were significantly associated. Similarly, Singer et al. (2022) found that nearly 8% of children with autism who did not have insomnia reported co-sleeping. In contrast, nearly 30% of children with both autism and insomnia reported co-sleeping (Singer et al., 2022). In addition, parents may choose to co-sleep with their children who have certain medical conditions -such as epilepsy- due to concerns about their safety (Accardo & Malow, 2015).

Although the literature on the relationship between sleep and ASD has yielded significant findings, many issues still need to be considered: (a) most studies have been conducted with small samples; (b) factors predicting sleep disorders have not been systematically investigated and are still unknown; and (c) few investigations have focused on co-sleeping.

Given this background, the present study aims to investigate sleep patterns in individuals with ASD. Specifically, we aim to describe the frequency and prevalence of sleep disturbances in a large Italian sample of children and adolescents, and to determine whether specific sociodemographic variables, psychological variables, and indices of cognitive and adaptive functioning can predict the presence/absence of sleep disorders. The secondary aim of the present work is to investigate the phenomenon of co-sleeping by assessing which specific factors characterize the group of children/adolescents with ASD who sleep with their parents.

## Method

### Participants and Procedure

Two hundred forty-two participants with ASD between the ages of 2 and 17 years were included in the study. The children and adolescents underwent neuropsychological and clinical assessment at the Child and Adolescent Neuropsychiatry Unit of the Bambino Gesù Children’s Hospital in Rome between January 2021 and December 2022. Specifically, participants were selected among individuals who received a diagnosis of ASD according to DSM—5 criteria (APA, 2013), performed by a multidisciplinary team including a senior child psychiatrist and an experienced clinically trained research child psychologist. All enrolled participants had a primary diagnosis of ASD without established genetic syndromes (see Table 1 for demographic, cognitive, and psychopathological measures of participants).

**Table 1:** Characteristics of participants (N = 242)

	N (%) or Mean (SD)
Age	5.03 ± 3.15
Range	2–17 years
Sex	
Males	194 (80.2)
Females	48 (19.8)
IQ/DQ	67.95 ± 23.03
ABAS-II	
General adaptive composite	61.23 ± 17.15
Conceptual domain	64.44 ± 16.35
Social domain	65.71 ± 15.80
Practical domain	65.56 ± 17.15
ADOS-2*	
Reciprocal social interaction	12.07 ± 4.16
Repetitve behaviors	3.32 ± 1.56
Calibrated severity score	6.38 ± 1.59
Total	15.40 ± 5.18
CARS2**	34.25 ± 5.66
ADI-R***	
Reciprocal social interaction	13.76 ± 4.09
Communication	9.15 ± 3.02
Repetitve behaviors	4.39 ± 1.74
Developmental abnormalities	4.37 ± 0.98
CBCL	
Internalizing problems	60.36 ± 9.99
Clinically relevant (≥ 64)	109 (45)
Borderline (60–63)	34 (14)
Absence (< 60)	99 (40.90)
Externalizing problems	55.47 ± 9.70
Clinically relevant (≥ 64)	165 (68.20)
Borderline (60–63)	28 (11.60)
Absence (< 60)	49 (20.20)
Total problems	59.37 ± 11.19
Clinically relevant (≥ 64)	126 (52.10)
Borderline (60–63)	35 (14.50)
Absence (< 60)	81 (33.50)
PSI	
Parental distress	55.40 ± 32.76
Parent–child dysfunctional interaction	70.24 ± 26.87
Difficult child	66.54 ± 29.65
Total Stress	66.11 ± 31.46

*M* Mean, *SD* standard deviation, *IQ* intelligence quotient, *DQ* global developmental quotient, *ABAS-II* adaptive behavior assessment system-second edition *ADOS-2* autism diagnostic observation schedule-2, *CARS2* childhood autism rating scale second edition, *ADI-R* autism diagnostic interview-revised, *CBCL* child behavior checklist, *PSI* parenting stress index-short form

^*^N = 167

^**^N = 75

^***^N = 241

Only participants who completed the assessment with all required instruments assessing intellectual abilities, daily living skills, ASD, parental stress, and behavioral and emotional symptoms (see Measures) were included in the final group.

Exclusion criteria were: the presence or clinical suspicion of neurological disorders (e.g., epilepsy, cerebral palsy, stroke, meningitis, encephalitis, brain tumors, cerebrovascular disorders), and a language barrier that prevented parents from completing the questionnaire.

At the end of the diagnostic procedure, the Sleep Disturbance Scale for Children (SDSC; Bruni et al., 1996; Romeo et al., 2013, 2021a) was administered to caregivers to assess the presence/absence of sleep disturbance in participants with ASD. In the second year of the study, the presence of co-sleeping was assessed by physicians in the last part of recruited children and adolescents with ASD. Namely, only a subset of 146 participants had the opportunity to respond to the ad hoc question about co-sleeping.

All caregivers were informed of the procedures and aims of the study and provided their written informed consent. The study has been approved by the Institutional Review Board of the Department of Psychology (#0002577) and was conducted in accordance with the Declaration of Helsinki.

### Measures

#### Cognitive Assessment

Cognitive development was assessed using a variety of instruments, depending on language ability and attentional resources:

- The Leiter International Performance Scale-Third Edition (Leiter-3; Roid et al., 2016) allows us to obtain a nonverbal intelligence quotient (IQ), independent of language and formal schooling. The complete IQ composite is based on four subtests (Figure Ground, Form Completion, Classification and Analogies, and Sequential Order);
- The Griffiths Scales of Child Development, 3rd Edition (Griffiths III; Green et al., 2016) provides a measure of children’s development in five domains: Foundations of Learning, Language and Communication, Eye and Hand Coordination, Personal-Social-Emotional, and Gross Motor. The average of the quotients of the five subscales provides a Global Developmental Quotient (DQ);
- The 36-item Colored Progressive Matrices (CPM; Raven, 2008), which assesses the ability to form perceptual relations and reason by analogy, independent of language and formal schooling, yielding a total IQ;
- The Wechsler Intelligence Scale for Children-fourth edition (WISC-IV; Wechsler, 2012) was used in our study in the absence of language problems. The instrument consists of 10 core subtests: Block Design, Similarities, Digit Span, Picture Concepts, Coding, Vocabulary, Letter–Number Sequencing, Matrix Reasoning, Comprehension, and Symbol Search. WISC-IV administration provides a global IQ.

The IQ of the Leiter-3, CPM, and Wisc-IV and the DQ of the Griffiths III were included in the present study.

#### Adaptive Functioning Assessment

Adaptive functioning was assessed using the Adaptive Behavior Assessment System-Second Edition Parent Form (ABAS-II; Harrison & Oakland, 2014). The ABAS-II is a questionnaire for caregivers about general adaptive abilities. It provides a general adaptive composite score (General Adaptive Composite) and three specific composite scores: Conceptual domain, Social domain, and Practical domain. Each composite score [Mean (M) = 100, Standard Deviation (SD) = 15] was considered in the present study.

#### Autism Diagnostic Observation Schedule, Second Edition (ADOS-2)

The Autism Diagnostic Observation Schedule-2 (ADOS-2; Lord et al., 2013) is the gold standard instrument for assessing ASD symptoms: communication, social interaction, play or imaginative use of materials, restricted/repetitive behaviors, or interests. In the present study, raw scores for Reciprocal social interaction, Repetitive behaviors, Calibrated Severity Score (CSS), and Total score were considered.

#### Childhood Autism Rating Scale Second Edition (CARS2)

The Childhood Autism Rating Scale Second Edition (CARS2; Schopler et al., 2014) is a 15-item behavioral rating scale designed to identify ASD and quantitatively describe the severity of the disorder. This instrument was administered to N = 75 participants who were not assessed with the ADOS-II. Specifically, CARS2 was employed to reduce physical contact with patients during the pandemic period to limit the spread of COVID-19. The items are as follows: I. Relating to People; II. Imitation; III. Emotional Response; IV. Body Use; V. Object Use; VI. Adaptation to Change; VII. Visual Response; VIII. Listening Response; IX. Taste, Smell, and Touch Response and Use; X. Fear or Nervousness; XI. Verbal Communication; XII. Nonverbal Communication; XIII. Activity Level; XIV. Level and Consistency of Intellectual Response; and XV. General Impressions. Each item is scored from 1 (no pathology) to 4 (severe pathology) at 0.5 intervals. Raw scores were used in the present study.

#### Autism Diagnostic Interview-Revised (ADI-R)

The Autism Diagnostic Interview-Revised (ADI-R; Rutter & Lord, 2005) is a 93-item standardized diagnostic interview administered to the caregiver to obtain information about: (a) qualitative abnormalities in reciprocal social interaction (domain A); (b) qualitative abnormalities in communication (domain B); (c) restricted, repetitive and stereotyped behaviors (domain C); and developmental abnormalities evident at or before 36 months of age (domain D). Raw scores were used in the present study.

#### Child Behavior Checklist (CBCL)

The Child Behavior Checklist (CBCL; Achenbach & Rescorla, 2001) is a well-established and widely used parent-completed measure of emotional, behavioral, and social problems in children and adolescents aged 1.5–18. Specifically, we used the two different versions of the CBCL (1.5–5 years or 6–18 years) depending on the age of the participants. The CBCL 1.5–5 consists of 100 problem items identified on several subscales, including Emotionally Reactive, Anxious/Depressed, Somatic Complaints, Withdrawn, Sleep Problems, Attention Problems, and Aggressive Behavior. In addition, scores can be obtained for Internalizing, Externalizing, and Total Problems. The Internalizing domain is a broad measure of emotional problems. It is an aggregate of anxiety and depression symptoms that subsumes four more narrowly focused syndrome scales: Emotionally Reactive, Anxious/Depressed, Somatic Complaints, and Withdrawn. The Externalizing domain is an aggregate measure of behavioral problems and includes Attention Problems and Aggressive Behavior. The Total Problems score quantifies the overall level of emotional and behavioral problems based on responses to all CBCL items.

In the CBCL 6–18, the 113-item scale is also divided into several subscales, namely Withdrawn/Depressed, Somatic Complaints, Anxious/Depressed, Rule- Breaking Behavior, Social Problems, Thought Problems, Attention Problems, and Aggressive Behavior. As for the CBCL 1.5–5, scores can be obtained for Internalizing, Externalizing, and Total Problems. The Internalizing domain subsumes three syndrome scales: Anxious/Depressed, Withdrawn/Depressed, and Somatic Complaints. The Externalizing domain includes the Rule-Breaking Behavior and Aggressive Behavior syndrome scales. Total Problems is based on responses to all CBCL items including those on the three remaining syndrome scales: Social Problems, Thought Problems, and Attention Problems.

In the current study, Total Problems, Internalizing Problems, and Externalizing Problems scores -that are overlapped in the two versions-were used as an estimate of behavioral and emotional problems. Raw scores are converted to T-scores, and according to the normative data of the CBCL, a T-score ≤ 59 indicates nonclinical symptoms, a T-score between 60 and 63 indicates that the child is at risk for problem behavior, and a T-score ≥ 64 indicates clinical symptoms.

#### Parental Stress Assessment

The Parenting Stress Index-Short Form (PSI; Abdin, 2016) assesses caregivers’ stress levels. The test assesses three domains: Parental distress, Parent–child dysfunctional interaction, and Difficult child. The sum of all questions results in a Total Stress score. Raw scores were converted to percentile scores and included in the present study.

#### Sleep Measures

The Sleep Disturbance Scale for Children (SDSC; Bruni et al., 1996) is a 26-item questionnaire that assesses the occurrence of sleep disorders during the past 6 months. The original version of the SDSC has been validated for children and adolescents between the ages of 6 and 18 and includes six subscales representing the most common areas of sleep disorders in childhood and adolescence: Disorders of Initiating and Maintaining Sleep (DIMS); Sleep Breathing Disorders (SBD); Disorders of Arousal (DA) such as sleepwalking, sleep terrors, nightmares; Sleep–Wake Transition Disorders (SWTD) such as hypnic jerks, rhythmic movement disorders, hypnagogic hallucinations, nocturnal hyperkinesia, bruxism; Disorders of Excessive Somnolence (DOES); Sleep Hyperhidrosis (SHY). In the current study, we also administered the SDSC adaptation for 6–36 months (Romeo et al., 2021a) and 3–6 years (Romeo et al., 2013) to parents, depending on the age of the participants. The infant version of the SDSC included 19 items and the following subscales: Disorders of Initiating Sleep (DIS); Disorders of Maintaining Sleep (DMS); Sleep Breathing Disorders (SBD); Parasomnias (PAR; Disorders of Arousal and Sleep Wake Transition); Disorders of Excessive Somnolence (DOES); Sleep Hyperhidrosis (SHY).

The SDSC version for children aged 3–6 years had 26 items and the following 6 subscales: Disorders of Initiating and Maintaining Sleep (DIMS); Sleep Breathing Disorders (SBD); Parasomnias (PAR; Disorders of Arousal and Sleep Wake Transition); Disorders of Excessive Somnolence (DOES); Sleep Hyperhidrosis (SHY); Nonrestorative Sleep (NRS).

The SDSC provides a T-score for each subscale and a total score (SDSC Total Score). A T-score of 60 or higher indicates a high or clinically significant score, indicating the presence of a sleep disorder.

In addition to the SDSC Total Score, the following sleep disturbances were included in the analyses: Sleep Breathing Disorders (SBD); Disorders of Excessive Somnolence (DOES); Sleep Hyperhidrosis (SHY); Disorders of Initiating and Maintaining Sleep (DIMS; DIS and DMS); Parasomnias (PAR; DA and SWTD).

In the second year of our data collection (2022), we decided to systematically collect information on co-sleeping, as it was often spontaneously reported during interviews with parents. Co-sleeping was investigated in a subgroup of 146 children. An ad hoc double-choice question was included at the end of the SDSC questionnaire to ask parents whether they shared a room or bed with their child or whether the child slept in a separate room.

### Statistical Analysis

First, descriptive analyses were performed on the following variables: age, sex, cognitive abilities (IQ/DQ), adaptive functioning (ABAS-II), symptoms of ASD (ADOS-2; ADI-R; CARS2), emotional and behavioral scores (CBCL), and parental stress (PSI).

In addition, the frequency and percentage of each sleep disorder assessed by the SDSC were calculated. To better differentiate between the presence and absence of sleep disorders, participants were divided into two groups based on their T-scores. The first group consisted of individuals with a T-score of 60 or higher on the sleep disorder measure, indicating the presence of a sleep disorder. The second group consisted of individuals with T-scores below 60 and a total absence of a sleep disorder.

A binary multivariable logistic regression was then computed to explore the best explanatory variables of sleep disorders (presence or absence; dependent variable), considering as independent variables: age, sex, IQ/DQ, three domains of the ABAS-II (Conceptual, Social, and Practical), CBCL (Internalizing Problems and Externalizing Problems), and the Total Stress score of the PSI.

The variables were entered into the model simultaneously. Multicollinearity among the independent variables was assessed using the variance inflation factor (VIF). For all predictors, the VIF was less than 4 (moderate correlation).

Finally, to better understand the “Co-sleeping” phenomenon, we performed a univariate ANOVA to test the differences between Co-sleepers and Not Co-sleepers, considering age, IQ/DQ, ABAS-II (General Adaptive Composite and Conceptual, Social, and Practical domains), CBCL (Total Problems, Internalizing Problems, and Externalizing Problems), PSI (Total Stress score, Parental distress, Parent–child dysfunctional interaction, and Difficult child) as dependent variables.

All analyses were conducted using the Statistical Package for Social Sciences (SPSS) version 25.0 and MATLAB R2019. The statistical significance was set at p < 0.05.

## Results

### Characteristics of Participants

Means and standard deviations for continuous variables and frequencies and percentages for categorical variables are shown in Table 1. The final group (N = 242) had a mean age of 5.03 ± 3.15 years. Most participants were male (80.2%). Regarding the cognitive assessment, the participants showed a mean IQ/DQ of 67.95 ± 23.03. The ABAS-II General Adaptive Composite score (M = 61.23 ± 17.15) was in the same range as the mean IQ/DQ score (two SD below the M = 100 ± 15). As for the co-occurring behavioral and emotional symptoms assessed with the CBCL, 45% of the group had Internalizing Problems, approximately 68% had Externalizing Problems, and more than half of the participants (52.10%) had clinically relevant Total Problems. The mean Total Stress score for the whole sample, as assessed by the PSI, is around the 66th percentile, indicating moderate distress. All other measures of ASD symptoms (ADOS-2, CARS2, ADI-R) showed clinical mean values.

### Distribution of Sleep Disorders

The frequencies and percentages of each sleep disorder and the mean and SD of the total score for all 242 children and adolescents with ASD are shown in Table 2. Approximately 33% of participants had an elevated or clinically relevant global score for sleep disorders (≥ 60). Regarding specific sleep disturbances explored in the overall sample, we found that (a) the most common sleep disorder in our group was sleep onset and sleep maintenance problem (about 41%); (b) about 15% of the children and adolescents reported elevated or clinically significant sleep breathing disorders; (c) 23% of the participants had a high score on the scale measuring excessive sleepiness; (d) 24% of the children and adolescents reported an above-average hyperhidrosis score; and (e) 20% of participants had a high score on the parasomnias scale.

**Table 2:** Distribution of sleep disorders (N = 242)

Sleep breathing disorders	
High scores/clinically relevant (≥ 60)	36 (14.9)
Absence (< 60)	206 (85.1)
Excessive Somnolence	
High scores/clinically relevant (≥ 60)	55 (22.7)
Absence (< 60)	187 (77.3)
Hyperhidrosis	
High scores/clinically relevant (≥ 60)	58 (24.0)
Absence (< 60)	184 (76.0)
Disorders of initiating and maintaining sleep	
High scores/clinically relevant (≥ 60)	101 (41.7)
Absence (< 60)	141 (58.3)
Parasomnias	
High scores/clinically relevant (≥ 60)	49 (20.2)
Absence (< 60)	193 (79.8)
SDSC total score	
Total score (M ± SD)	56.62 ± 12.94
High scores/clinically relevant (≥ 60)	81 (33.5)
Absence (< 60)	161 (66.5)

*M* Mean, *SD* standard deviation, *SDSC* sleep disturbance scale for children

### Predictors of Sleep Disorders

**Fig. 1:**
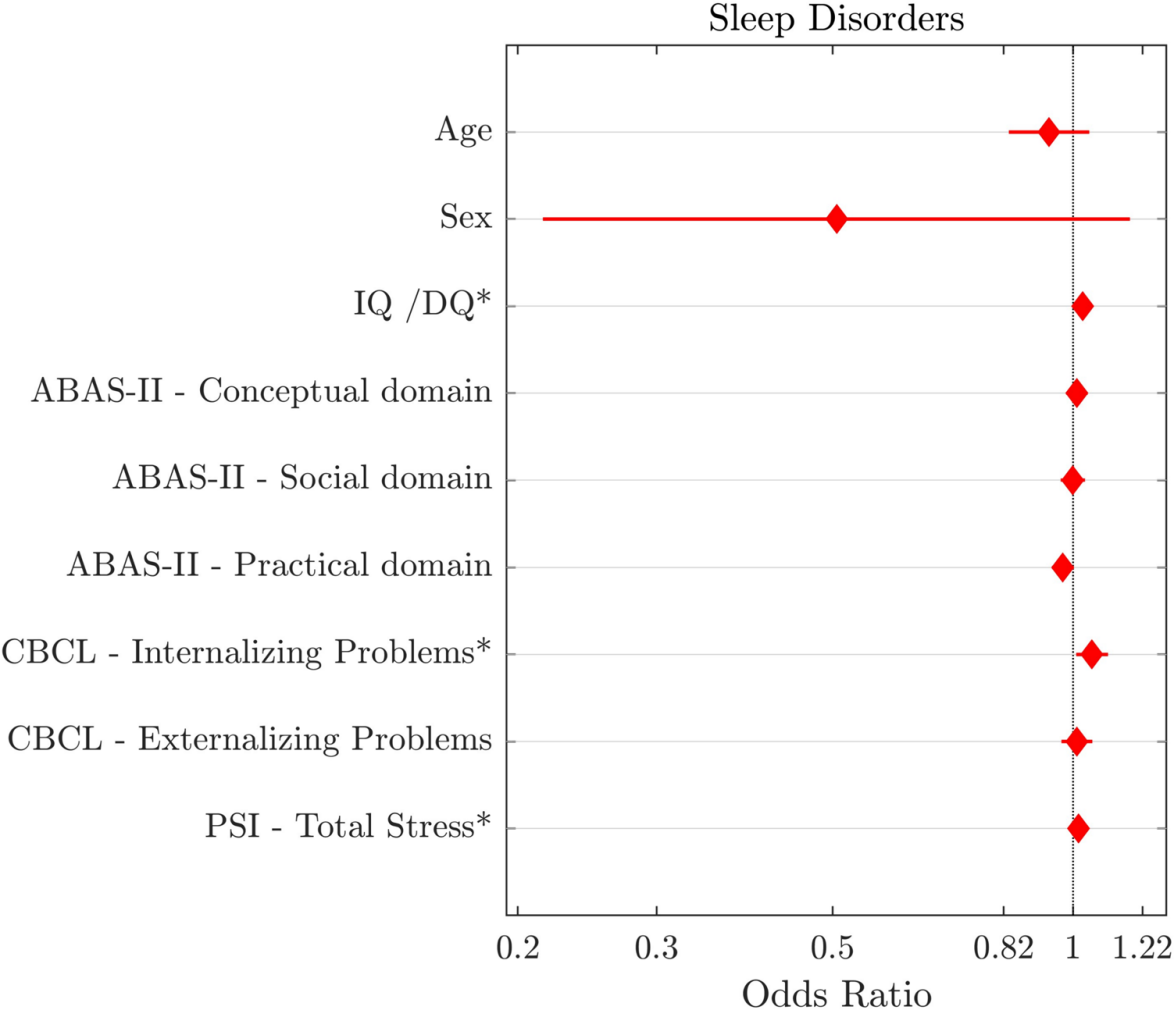
Multiple binary logistic regression model with sleep disorders (presence or absence) as dependent variable and socio-demographic variables, cognitive functioning, adaptive functioning, psychological measures as predictors (N = 242). Graphic representation of odds ratio and relative 95% confidence intervals for each predictor: age, sex (reference: male), Intelligence Quotient (IQ)/Global Developmental Quotient (DQ), *ABAS-II* adaptive behavior assessment system-second edition, *CBCL* child behavior checklist and *PSI* parenting stress index-short form. Independent significant predictors for each outcome are marked with asterisks (p < 0.05)

The binary logistic regression model with sleep disorders (presence or absence) as the dependent variable was significant (likelihood ratio: chi-squared = 56.445; p < 0.001; Negelkerke’s *R*^2^ = 0.289). The results (see Fig. 1 and Table 3) showed that IQ/DQ (p = 0.003; odds ratios [OR], 1.028; 95% confidence intervals [CI], 1.010–1.047), Internalizing Problems (p = 0.02; OR, 1.056; CI, 1.009–1.106), and the parental Total Stress (*p* = 0.017; OR, 1.016, CI, 1.003–1.029) were significant predictors of sleep disorders. Specifically, higher IQ/DQ, greater Internalizing Problems, and higher parental stress predict the presence of sleep disorders in children and adolescents with ASD.

**Table 3:** Results of binary logistic regression with sleep disorders (presence or absence) as the dependent variable and socio-demographic variables, cognitive functioning, adaptive functioning, and emotional and behavioral symptoms as predictors (N = 242)

Covariates	OR	p	95% CI	
			Lower bound	Upper bound
Age	0.933	0.241	0.831	1.048
Sex				
Females	0.506	0.114	0.217	1.178
Males	Ref			
IQ/DQ	**1.028**	**0.003**	**1.010**	**1.047**
ABAS-II—conceptual domain	1.011	0.476	0.980	1.043
ABAS-II—social domain	0.999	0.977	0.965	1.035
ABAS-II—practical domain	0.971	0.063	0.941	1.002
CBCL—internalizing problems	**1.056**	**0.020**	**1.009**	**1.106**
CBCL—externalizing problems	1.011	0.635	0.967	1.057
PSI—total stress	**1.016**	**0.017**	**1.003**	**1.029**

*OR* Odd ratio, *CI* confidence interval, *Ref* reference, *IQ* intelligence quotient, *DQ* global developmental quotient*, ABAS-II* adaptive behavior assessment system-second edition, *CBCL* child behavior checklist, *PSI* parenting stress index-short form

Significant values are in bold

### Characteristics of Co-sleepers

We found that 59.6% of the 146 individuals with ASD in whom we studied Co-sleeping shared bed with their parents (N = 87, 2–12 years). The prevalence in Italian typically developing subjects (N = 901, 6–12 years) was 5% (Cortesi et al., 2004).

Statistical comparisons showed that Co-sleepers had lower age (F = 7.091; p = 0.009), lower IQ/DQ (F = 8.624; p = 0.004), lower adaptive functioning (ABAS-II) in the Conceptual domain (F = 4.391; p = 0.038) and the General Adaptive Composite (F = 5.259; p = 0.023) and more total problems as assessed by CBCL (F = 4.051; p = 0.046) than the No Co-sleepers group. Means and SDs for each variable, F-values, and p-values are reported in Table 4.

**Table 4:** Results of the univariate ANOVA comparing Co-sleepers (N = 87) vs. No Co-sleepers (N = 59)

	Media (DS)	F-values	p-values
Age			
No co-sleepers	5.12 (2.79)	**7.091**	**0.009**
Co-sleepers	4.13 (1.69)		
IQ/DQ			
No co-sleepers	75.61 (19.95)	**8.624**	**0.004**
Co-sleepers	65.51 (20.70)		
ABAS-II—general adaptive composite			
No co-sleepers	65.02 (18.53)	**5.259**	**0.023**
Co-sleepers	58.31 (16.49)		
ABAS-II—conceptual domain			
No Co-sleepers	67.83 (17.54)	**4.391**	**0.038**
Co-sleepers	61.64 (16.07)		
ABAS-II—social domain			
No Co-sleepers	67.59 (17.18)	1.110	0.294
Co-sleepers	61.64 (15.19)		
ABAS-II—practical domain			
No Co-sleepers	68 (19.87)	2.971	0.087
Co-sleepers	63 (15.13)		
CBCL—internalizing problems			
No Co-sleepers	59.63 (11.22)	2.150	0.145
Co-sleepers	62.20 (9.78)		
CBCL—externalizing problems			
No co-sleepers	54.75 (9.53)	3.003	0.085
Co-sleepers	57.47 (9.19)		
CBCL—total problems			
No Co-sleepers	58.34 (11.15)	**4.051**	**0.046**
Co-sleepers	62.17 (11.39)		
PSI—parental distress			
No Co-sleepers	49.37 (31.17)	3.496	0.087
Co-sleepers	59.90 (34.78)		
PSI—parent–child dysfunctional interaction			
No Co-sleepers	66.75 (30.07)	1.591	0.209
Co-sleepers	72.47 (24.55)		
PSI—difficult child			
No Co-sleepers	63.83 (33.34)	2.714	0.102
Co-sleepers	71.95 (26.17)		
PSI—total stress			
No co-sleepers	62.03 (33.91)	2.299	0.132
Co-sleepers	70.11 (29.94)		
SDSC—total score			
No co-sleepers	59.68 (12.43)	0.003	0.959
Co-sleepers	59.79 (13.85)		

*IQ* Intelligence quotient, *DQ* global developmental quotient, *ABAS-II* adaptive behavior assessment system-second edition, *CBCL* child behavior checklist, *PSI* parenting stress index-short form

Significant differences in bold

## Discussion

### Sleep Disorders in Autism

The current study investigated the relationship between sleep disorders and different characteristics of 242 Italian children and adolescents with ASD. First, we examined the distribution of sleep disturbances in the whole group. According to the previous literature (Galli et al., 2022; Singer et al., 2022; Veatch et al., 2017), we found that sleep onset and sleep maintenance problems were the most common disorders, affecting approximately 41% of our participants. Similarly, Galli et al. (2022) showed that 57% of Italian children with ASD (N = 100) suffered from insomnia symptoms. Difficulties in falling asleep or staying asleep are common in individuals with ASD, as reported in several studies using parental reports (Gail Williams et al., 2004; Krakowiak et al., 2008; Liu et al., 2006; Malow & McGrew, 2008). Studies using objective methods (i.e., actigraphy, polysomnography) have also partially confirmed these findings (Malow et al., 2006; Souders et al., 2009). The actigraphy results showed that 66.7% of the children with ASD had disturbed sleep. Specifically, the ASD group had longer sleep latency (more than 30 min) and longer wake episodes than the healthy controls. In addition, a polysomnographic study found that children with ASD, who were rated as poor sleepers by their parents, exhibited lower sleep efficiency and longer sleep latency compared to both ASD children evaluated as good sleepers and the control group (Malow et al., 2006).

It is worth noting that specific symptoms typically found in individuals with ASD, such as bedtime rituals, repetitive activities, sensory dysregulation (Mazurek & Petroski, 2015; Souders et al., 2009; Wiggs & Stores, 2004), phobias (Leyfer et al., 2006), pharmacological treatment (Mazzone et al., 2018; Panju et al., 2015) could interfere with sleep onset and sleep continuity.

Importantly, discriminating between circadian alterations and insomnia can be challenging in ASD since both biological and environmental variables may contribute to these disturbances (Carmassi et al., 2019). Our sample also included adolescents, and for this reason, it is necessary to consider the potential impact of developmental changes in the circadian regulation of sleep that occurs with delayed melatonin rhythms in this population (Alfonsi et al., 2020; Uccella et al., 2023). In fact, adolescents show a well-documented tendency to eveningness. In particular, during puberty, the natural tendency to remain active until late at night and to wake up late in the morning emerges, and the circadian system undergoes a lengthening of its period, resulting in a delay in the onset of sleep (Alfonsi et al., 2020). To correctly diagnose insomnia, the delayed sleep phase disorder should be excluded (de Zambotti et al., 2018). However, some authors have noted a partial overlap (approximately 50%) between delayed sleep phase disorder as defined by ICSD-3 and insomnia defined by quantitative criteria (e.g., difficulty initiating and maintaining sleep, tiredness or sleepiness occurring at least three times per week, and sleep onset latency and/or wake after sleep onset greater than 30 min) (de Zambotti et al., 2018; Sivertsen et al., 2013). In other words, only a systematic and prospective assessment of sleep through sleep diaries over prolonged periods could improve the differentiation of the circadian alteration typical of delayed sleep phase from insomnia disorder.

According to previous studies (Díaz-Román et al., 2018; Liu et al., 2006; Romeo et al., 2021b), we also found that approximately 20% of individuals with ASD reported high scores in parasomnia, hyperhidrosis, and excessive somnolence scales. Some studies have highlighted that nocturnal sweating is a comorbid symptom of various sleep disorders in both children with typical development (Chang & Chae, 2010; So et al., 2012) and neurodevelopmental disorders (Romeo et al., 2021b; Shelton & Malow, 2021). Interestingly, a sample of 747 children with typical development and nocturnal sweating reported an association with obstructive sleep apnea (OSA), insomnia, and parasomnias (So et al., 2012). Specifically, increased sweating may result from the nocturnal movements, frequent postural changes, and restless sleep observed in parasomnia, OSA and insomnia, respectively (Chang & Chae, 2010; Shelton & Malow, 2021; So et al., 2012), with concomitant autonomic nervous system activation and dysregulation of neural control of the skin (So et al., 2012). In this view, nocturnal sweating may be indicative of underlying sleep disturbances and could alert clinicians to provide early detection and treatment of sleep problems in the pediatric population (So et al., 2012).

In addition, we found that approximately 15% of children and adolescents with ASD reported elevated or clinically significant sleep breathing disorders. It should be noted that the prevalence of this disturbance in the pediatric population ranges from 2 to 5% (Kaditis et al., 2016; Saran et al., 2024). Our finding is consistent with previous research suggesting that individuals with ASD may be at increased risk for sleep apnea and other breathing-related sleep disturbances (Gail Williams et al., 2004; Hirata et al., 2016; Mutluer et al., 2016; Tomkies et al., 2019). However, findings regarding sleep disordered breathing in children with ASD are not homogeneous (Limoges et al., 2005; Malow et al., 2006). It should be noted that some co-occurring factors may increase the rate of respiratory problems, such as obesity and hypotonia in children with ASD (Curtin et al., 2010).

Notably, the proportion of individuals with ASD reporting high-risk or clinically relevant sleep disorders is lower in the present study than in others using different scales to assess sleep problems (for a review, see Carmassi et al., 2019). A recent investigation in an Italian sample (Romeo et al., 2021b) using the SDSC for preschool children showed that only 18% of children had a clinically relevant total score and 46% had an abnormal score on at least one subscale. Interestingly, although Romeo et al. (2021b) included only preschoolers, their mean SDSC total score (58.8 ± 13.2) is similar to our mean total score (56.62 ± 12.9).

Furthermore, most of the investigations with a high prevalence of sleep problems in autism (Irwanto et al., 2016; May et al., 2015; Taira et al., 1998; Tudor et al., 2015; Wiggs & Stores, 2004) included a significantly smaller sample than our study (≤ 88 individuals with autism). The small number of participants may be a critical flaw that greatly affects the prevalence data. Also, in some cases (e.g., Rzepecka et al., 2011) where the parent response rate was only 29%, we can hypothesize that the results may be affected by a “self-selection bias”. In other words, parents of children with ASD and co-occurring sleep problems may be more interested in participating in the protocol. In addition, other results may be influenced by the presence of other chronic medical comorbidities such as epilepsy or other neurodevelopmental disorders (e.g., Rzepecka et al., 2011; Wiggs & Stores, 2004).

### Explanatory Variables of Sleep Disorders in Children with ASD

We found that higher IQ/DQ, greater internalizing problems, and greater parental stress were significant predictors of sleep disorders. The current literature on the relationship between sleep and IQ reports mixed results, with some studies finding no association between sleep disturbance and intelligence (e.g., Mayes & Calhoun, 2009). Consistent with our findings, Richdale and Prior (1995) found that children with ASD with higher cognitive scores (IQ > 55) had more severe sleep problems compared to the group with lower scores (IQ < 55) and a control group. Similarly, Couturier et al. (2005) reported higher sleep problems in children with ASD with average IQ. A large study of 1583 children found that IQ positively predicted sleep anxiety. The authors suggested that children with Asperger’s disorder were more likely to develop sleep problems (Hollway et al., 2013). Conversely, other studies reported that an IQ < 70 was associated with increased difficulty with sleep onset and nighttime awakenings, as well as shorter sleep duration (Didden et al., 2002; Giannotti et al., 2011; Krakowiak et al., 2008; Miano et al., 2007).

The reasons for the association between IQ and sleep disturbances are not fully understood. On the one hand, it could be hypothesized that the clinical profile of individuals with ASD without intellectual disability is associated with better communication skills, facilitating their sleep-related complaints to parents. On the other hand, caregivers may pay more attention to sleep problems when children have average or higher cognitive scores. Intellectual disability might be a factor that exacerbates the clinical condition in ASD, and sleep problems are likely to remain unconsidered among other multiple problems. In addition, children with higher IQ scores may be more prone to rumination and worries related to their social problems (Gotham et al., 2014), which may also affect sleep (You et al., 2021). In other words, children with average or high IQ might be more aware of their impaired social experiences and report more feelings of fear and anxiety.

Notably, electrophysiological (EEG) findings indicated that children with ASD having an IQ ≥ 80 showed impaired activation of neural networks responsible for REM sleep control (Daoust et al., 2004). In fact, the authors showed that children with HFA had lower beta activity during REM sleep over occipital regions than controls (Daoust et al., 2004). It is worth noting that REM sleep instability (Riemann et al., 2012) could explain the presence of sleep disturbances—especially some insomnia symptoms—in HFA. In particular, some evidence emphasized that poor REM sleep characterized by arousals and micro-awakenings could induce the perception of non-restorative sleep, which -at the same time- could affect emotional regulation processes (Riemann et al., 2012).

Along this vein, we found that greater internalizing problems were significantly associated with sleep disturbances. This is consistent with several findings highlighting a strong relationship between anxiety or other internalizing behaviors and disturbed sleep in ASD (Hollway et al., 2013; Limoges et al., 2005; Patzold et al., 1998). The available literature shows that anxiety and related hypervigilance/arousal are common in children and adolescents with ASD, predisposing them to sleep problems (Mazurek & Petroski, 2015; Nadeau et al., 2015). We must also emphasize that the internalizing subscale of the CBCL version for subjects aged 1.5–5 years includes an assessment of sleep problems. This may partly explain the association found between internalizing problems and sleep disorders in our sample.

We also found that parental stress was a significant predictor of sleep disorders. In addition to environmental factors (e.g., bedtime routines, daytime schedules), caregiver-related issues (e.g., family dynamics, maternal depression, parental stress) may often contribute to sleep disturbances in children with neurodevelopmental disorders (Jan et al., 2008). A recent study evaluating 177 children with ASD confirmed that parental stress was significantly higher when children were affected by sleep disturbances (Johnson et al., 2018). Similarly, a recent systematic review showed an association between overall caregiver mental health and sleep disturbances in children (Martin et al., 2019).

It could be hypothesized that a “vicious circle” may underlie the association between sleep problems and parenting stress in ASD. Indeed, altered sleep patterns in children and adolescents with ASD may contribute to parenting stress and poorer psychological well-being in parents, which in turn may lead caregivers to use less effective parenting strategies. In addition, as noted above, sleep problems may exacerbate the child’s behavioral difficulties (Schreck et al., 2004), thereby increasing parental distress. This bidirectional relationship makes this an important issue to consider for further studies and interventions.

### Co-sleeping in Autism

In a subset of participants with ASD, we also examined the phenomenon of co-sleeping. Interestingly, the prevalence of co-sleeping in an Italian sample of typically developing children was 5% (Cortesi et al., 2004), while in the subsample examined in the current study it was significantly higher, reaching almost 60%.

We showed that Co-sleepers are younger and have lower IQ/DQ, lower adaptive functioning, and lower psychological well-being than No Co-sleepers. The lower frequency of co-sleeping in “older” individuals with ASD is consistent with other findings (e.g., Liu et al., 2006). Furthermore, this finding is partially consistent with data from typically developing individuals, as the prevalence of co-sleeping decreases significantly with age throughout childhood (Cortesi et al., 2004).

Also, we found that individuals with more co-occurring problems tended to sleep with their parents. Accordingly, Patzold et al. (1998) found that problematic daytime behavior was associated with co-sleeping. In addition, findings on children and adolescents without ASD showed higher rates of internalizing and psychiatric disorders among persistent co-sleepers (Santos et al., 2016).

Overall, the lower IQ/DQ scores, combined with repetitive behaviors, may lead caregivers to believe that their children need more care at night. In fact, restricted and stereotyped behaviors could make it difficult to separate the child from the parents’ room (Liu et al., 2006). With this in mind, we hypothesized, according to Cortesi et al. (2004), that co-sleeping may reflect a parental strategy to cope with sleep problems, reducing their manifestations in children with more severe conditions. In this sense, previous studies have suggested that co-sleeping is prevalent among children with chronic conditions such as neurological disorders, cerebral palsy, and epilepsy (Jacquier & Newman., 2017; Larson et al., 2012). In such cases, the practice of parent–child co-sleeping is primarily reactive and stems from the child’s nighttime care needs (Mörelius & Hemmingsson, 2014; Sidhoum et al., 2019).

The IQ differences between Co-Sleepers and No Co-sleepers also need to be considered, as individuals who do not report co-sleeping have -at the same time- higher IQs. As discussed earlier, individuals with higher IQ (a) may be more likely to express sleep-related difficulties and (b) their parents may have a greater tendency to take sleep difficulties into account. Thus, the difference between the IQs of Co-sleepers and No Co-sleepers could partly explain the lack of variation between the two groups in terms of sleep disturbances.

Overall, the relationship between co-sleeping and sleep quality is far from being linear. In fact, it may be bidirectional, and the costs or benefits of this practice remain to be determined. The lack of differences between Co-sleepers and No Co-sleepers in terms of sleep disorders is partly inconsistent with the current literature, which suggest that co-sleeping is a risk factor for poorer sleep quality (Garrido et al., 2024; Köse et al., 2017; Liu et al., 2006; Singer et al., 2022). However, there are no longitudinal data to support this hypothesis.

On the one hand, it is possible that families choose to co-sleep because their children have sleep problems. It should be noted that among typically developing subjects, one of the main reasons for initiating co-sleeping was problematic bedtime sleep behavior or disruptive nighttime behavior (72%; Cortesi et al., 2004). On the other hand, co-sleeping may be a protective factor in the development of sleep problems in children with autism. This hypothesis could be consistent with our negative finding of no association between parental stress and co-sleeping. A recent study investigating co-sleeping in a large sample aged 0.5–21 years with various medical comorbidities revealed that co-sleeping was reassuring and comforting for 27% of parents (Sidhoum, et al., 2019). Notably, two-thirds of caregivers reported a positive relationship between the practice of co-sleeping and their own sleep quality (Sidhoum, et al., 2019).

Finally, other variables may influence the relationship between co-sleeping and child outcomes. For example, it may be influenced by cultural factors, parenting styles, and other contextual factors (Köse et al., 2017).

## Conclusions

According to the literature, we found that insomnia symptoms were the most commonly reported problem. In addition, we found that high IQ, great internalizing problems, and high caregiver stress were significantly associated with sleep problems. Moreover, in a subsample of 146 participants, we revealed that co-sleepers are younger, have lower adaptive and cognitive functioning, and have greater behavioral/psychological problems than No Co-sleepers, although no differences in sleep disorders were reported.

Our investigation had the advantage of better understanding many issues related to sleep problems in a large and well-selected Italian group of children and adolescents with ASD. However, the study had several limitations. First, the lack of a control group made it difficult to compare sleep parameters between participants with ASD and typically developing children. Second, we did not assess certain socio-demographic information, such as parental education, income, and minority status. We also recognize that co-sleeping may be influenced by these socio-cultural determinants. This is a relevant shortcoming of the current study that limits the generalizability of our findings.

Furthermore, the lack of actigraphic or polysomnographic (PSG) recordings did not allow for objective measures of sleep patterns. In addition, the lack of prospective measures (i.e., sleep diaries) may make it difficult to distinguish between delayed sleep phase disorder and insomnia in youth with ASD. Importantly, co-sleeping was only assessed in a sub-sample, providing a partial view of the phenomenon. Finally, the inclusion of children and adolescents with ASD of different ages prevented the use of the variables provided by the SDSC subscales as continuous, but only as categorical (in terms of presence/absence of sleep disorder). In fact, we used three different versions of the SDSC questionnaire, and this did not allow the researcher to consider the scores of each subscale, preventing more in-depth analyses of a single sleep disorder. Therefore, we believe that more research is needed to better understand the relationship between specific sleep problems and clinical, behavioral, and cognitive functioning in children and adolescents with autism. Additionally, to keep adequate statistical power we did not conduct separate analyses for different age groups. However, assessing sleep patterns across distinct age groups may deserve interest and should be taken into consideration in further investigations.

Future studies that combine prospective and objective methods (i.e., sleep diaries and actigraphic recordings) may help to better assess delayed sleep phase disorder or insomnia symptoms. In addition, on the one hand, our results on the prevalence of sleep disorders are influenced by the exclusion of neurological conditions such as epilepsy and cerebral palsy; on the other hand, we included participants regardless of whether they had reflux or gastrointestinal problems, which are highly correlated with sleep disorders (Al-Beltagi, 2021). In terms of future studies, it would indeed be valuable to investigate medical comorbidities as important and relevant predictors of sleep disturbance in ASD. This could provide further insights into the complex interplay between medical conditions and sleep disturbances in individuals with ASD, potentially leading to better management and interventions tailored to their specific needs.

Moreover, the phenomenon of co-sleeping certainly deserves further attention. It may be relevant a follow-up to investigate co-sleeping patterns in a larger sample controlling for age. More directly, providing longitudinal within-subjects data to check the development of sleep disorders over the years may be crucial to determine the relationship between sleep patterns and co-sleeping, and to evaluate the benefits and costs of this practice. Also, comparisons between a group with ASD versus a group with other non-ASD neurodevelopmental conditions and a control group should be provided.

Furthermore, home-PSG recordings and EEG topography should be performed to assess whether specific macro- and micro-structural sleep characteristics are altered in individuals with ASDs compared to a control group. In particular, based on EEG evidence of REM sleep disruption and occipital alterations in children with HFA (Doust et al., 2004), investigation of sleep microstructure and local sleep EEG features may be crucial to better understand the neural basis of arousal-related sleep processes in children with HFA. In this context, considering the association between parieto-occipital areas and REM-like dream activity (Scarpelli et al., 2019; Solms, 2000), which in turn is related to emotional regulation processes, it would be interesting to assess dream contents in children/adolescents with HFA (Daoust et al., 2008; Godbout et al., 2000).

Finally, individuals with and without sleep disorders, as assessed by parent-reported questionnaires, should be compared, controlling for IQ/DQ and adaptive functioning. We believe that a better understanding of the psychological and psychophysiological mechanisms underlying sleep problems in ASD may help to provide targeted interventions to improve the quality of their night’s rest, likely reducing some behavioral difficulties during wakefulness.
